# Photoporation-mediated spatial intracellular delivery of stem cell-derived cardiomyocytes

**DOI:** 10.1016/j.mex.2024.102548

**Published:** 2024-01-04

**Authors:** Laurens Léger, Chloë De Clercq, Jeffrey Aalders, Kiara Van Acker-Verberckt, Kevin Braeckmans, Jolanda van Hengel

**Affiliations:** a Medical Cell Biology Group, Department of Human Structure and Repair, Faculty of Medicine and Health Sciences, Ghent University, Ghent, Belgium; b Laboratory of General Biochemistry and Physical Pharmacy, Faculty of Pharmaceutical Sciences, Ghent University, Ghent, Belgium

**Keywords:** Intracellular delivery, Photoporation, Induced pluripotent stem cell-derived cardiomyocytes, Gold nanoparticle-sensitized photoporation

## Abstract

Human induced pluripotent stem cell-derived cardiomyocytes (iPSC—CMs) are promising candidates for disease modeling and therapeutic purposes, however, non-viral intracellular delivery in these cells remains challenging. Gold nanoparticle (AuNP)-sensitized photoporation creates transient pores in the cell membrane by vapor nanobubble formation, allowing diffusion of extracellular biomolecules. This non-viral technique was employed to test and optimize its distinct physical mode of action in iPSC—CMs. Photoporation optimization was aimed at achieving high delivery rates while minimizing cell death. Various AuNP concentrations, in conjunction with different laser fluences, were explored to facilitate the intracellular delivery of 10 kDa and 150 kDa FITC-labelled dextran as model macromolecules. Cardiomyocyte viability was assessed using the CellTiter-Glo® viability assay, while the delivery efficiency was quantified through flow cytometry. On 30 day-old cardiomyocytes, AuNP photoporation was able to yield ∼60 % delivery efficiency while maintaining a high cell viability (∼70 %). Overall, higher AuNP concentrations resulted in greater delivery efficiencies, albeit at the expense of lower cell viability. Finally, photoporation was capable of patterning a geometric shape, demonstrating its exceptional selective resolution in delivering molecules to spatially restricted regions of the cell culture. In conclusion, AuNP-photoporation exhibits considerable potential as an effective and gentle non-viral method for intracellular delivery in iPSC—CMs.

• AuNP-photoporation is a non-viral intracellular delivery method suitable for iPSC—CMs with high efficiency and cell viability
• This method is capable of spatially resolved intracellular delivery with excellent resolution

## Graphical abstract

**Figure:**
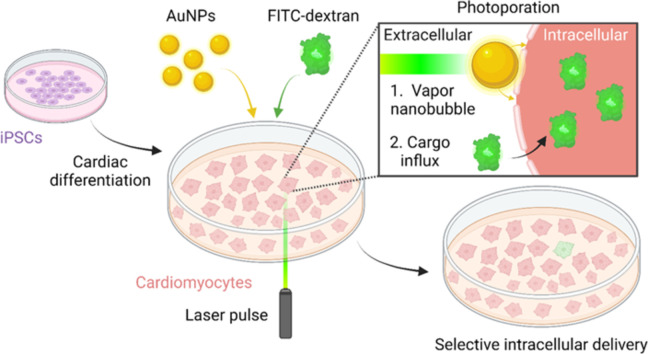

Specifications table

**Table:** 

Subject area:	Biochemistry, Genetics and Molecular Biology
More specific subject area:	Intracellular delivery
Name of your method:	Gold nanoparticle-sensitized photoporation
Name and reference of original method:	Photoporation: Xiong R, Raemdonck K, Peynshaert K, Lentacker I, De Cock I, Demeester J, De Smedt SC, Skirtach AG, Braeckmans K. Comparison of gold nanoparticle mediated photoporation: vapor nanobubbles outperform direct heating for delivering macromolecules in live cells. ACS Nano. 2014 Jun 24;8(6):6288–96 https://doi.org/10.1021/nn5017742
Resource availability:	The Lumipore™ photoporation platform can be found on https://trincebio.com/lumipore/

## Method details

The method outlined in this article is an adaptation of the previously published article describing vapor nanobubble (VNB) formation following gold nanoparticle (AuNP)-mediated photoporation to deliver macromolecules in cancer cells [1]. Also other cell types, including primary hippocampal neurons, insulin producing cells, keratinocytes, macrophages, human embryonic stem cells and mesenchymal stem cells have been successfully photoporated [1,2]. Optimization for human induced pluripotent stem cell-derived cardiomyocytes (iPSC—CMs) was undertaken in this study. High cardiomyocyte delivery efficiencies can be reached through viral means, such as adenoviral, adeno-associated viral (AAV), and lentiviral (LV) strategies [3]. Disadvantages include immunogenicity and insertional mutagenesis with LV transduction. Furthermore, iPSC-CMs require significantly higher viral AAV doses compared to primary cardiomyocytes, which can subsequently lead to reduced viability [3]. Non-viral options have been explored in the literature, with varying success. Magnetic nanoparticle-mediated transfection yielded 18 ± 2 % efficiency in iPSC-CMs [4]. Another study compared multiple non-viral transfection methods and showed that LipoSTEM, a liposomal based transfection method, was superior to transfect iPSC-derived cardiomyocytes with an efficiency of 35 to 55 % and a viability of 80 to 92 % [5]. This study also showed that the efficiency of other commonly used non-viral transfection methods was lower, with reported values of 17 ± 8.5 % for Transporter 5 Transfection Reagent (TR5) polyplex delivery, 12,7 ± 1 % in case of cationic polymer polyethylenimine (PEI-25), and 9 ± 3.2 % and 13 ± 3 % in case of Lipo2K and Lipo3K transfection respectively. Both studies employed flow cytometry, the golden standard, to assess transfection efficiency. When employing the cationic delivery reagent Viafect™, efficiencies up to 95 % with 66 % viability were reported, however, these values were solely based on microscopic observations [6].

Spatial control over intracellular delivery could be meaningful for a wide array of applications. Spatial and temporal regulation of gene expression is instrumental in developmental biology. For disease modelling purposes, spatial intracellular delivery could potentially open up new research avenues, allowing researchers to target subpopulations/regions and study protein-protein, protein-drug or cell-cell interactions. Screening could also be performed in a single dish by sequentially transfecting different regions of the cell culture. Regeneration and angiogenetic mechanisms are under careful spatial control of platelet-derived growth factor (PDGF) and vascular endothelial growth factor (VEGF), possibly explaining the disappointing results in clinical trials where administration of said compounds do not display the intricate spatial mode of action. Current approaches for cardiac spatial intracellular delivery are limited and mostly reliant on culturing the cells on 3D nanostructures rather than common cell culture dishes. Cao Y. and colleagues [7] developed a nanostraw-electroporation system, which could deliver eGFP mRNA to iPSC-CMs. A similar approach was employed by Cerea A. and colleagues [8], who integrated multi-electrode array technology with hollow nanostructures and microfluidics. Instead, photoporation allows to easily deliver compounds in selected cell culture areas in traditional culture dishes by controlled scanning of the activating laser beam, even with single cell resolution [9].

Several protocols are available for the cardiac differentiation of iPSCs, yielding cardiomyocytes promising for both fundamental research and pharmacological studies [10]. We have employed an adapted protocol by Burridge and colleagues [11] to obtain a pure population of iPSC—CMs, which is given in Supplementary File 1.

Gold nanoparticle-sensitized photoporation can be done directly on the cell culture plate with the differentiated cells, or the cardiomyocytes can be replated to other well formats (e.g. 96 wells) to achieve a higher throughput.

## iPSC-derived cardiomyocyte replating (optional)

Materials

- CELLSTAR® 96 wells (Greiner Bio One, cat. no. 655,180) OR other desired format
- Geltrex™ (Life Technologies, cat. no. A1413302)
- Multi Tissue Dissociation kit 3 (Miltenyi Biotec, cat. no. 130–110–204)
- Fetal Bovine Serum (FBS) (Life Technologies, cat. no. 10,500–064)
- Phosphate buffered saline (PBS) (Life Technologies, cat. no. 10,010,023)
- RPMI1640 + Glutamax + HEPES (Life Technologies, cat. no. 72,400–021)
- B27 supplement with insulin (Life Technologies, cat. no. 17,504–044)
- Penicillin/Streptomycin (100 U/ml Penicillin and 100 µg/ml Streptomycin, Life Technologies, cat. no. 15,140–122)
- DMEM/F-12 (Life Technologies, cat. no. 11,320–033)
- RevitaCell™ (Life Technologies, cat. no. A2644501)
- 15 ml plastic conical tubes
- Centrifuge

(1) Prepare a stock of cell culture medium (cardio culture) by supplementing RPMI1640 + GlutaMAX + HEPES with 1:50 B27 supplement with insulin and a 1:100 ratio of Penicillin/Streptomycin
(2) Prepare sufficient Geltrex-coated (1:100 diluted in DMEM/F-12, 100 µl/well) 96-well plates, depending on your application, by incubating them for 1 hour at 37 °C. Make sure to prepare everything in duplicate for delivery and viability assays
(3) Prepare sufficient of the Multi Tissue Dissociation kit 3 by mixing the supplied Buffer X with enzyme T (1:10)
(4) Wash the iPSC—CM dish with 115 µl/cm² PBS three times
(5) Add 115 µl/cm² of the Multi Tissue Dissociation kit 3 mixture to the wells
(6) Incubate for 10 min at 37 °C, 5 % CO_2_, 19 % O_2_
(7) Check the cells under the microscope to see if the cells are dissociated. Gently resuspend to obtain a single cell population
(8) Add two times the enzyme volume of cardio culture to inactivate the enzyme
(9) Take a small aliquot and count the cells using a method of your choice (e.g. trypan blue + Bürker counting chamber)
(10) Collect the cells and transfer to a 15 ml conical tube
(11) Centrifuge for 5 min at 200 g
(12) Remove supernatant and resuspend the iPSC—CMs in cardio culture medium supplemented with 20 % FBS and 1:100 RevitaCell
(13) Seed the iPSC—CMs in the Geltrex-coated 96-well plates at a density 20.000 cells/well (62.500 cells/cm²) and incubate at 37 °C, 5 % CO_2_, 19 % O_2_
(14) Replenish the medium every other day with cardio culture (no FBS or RevitaCell supplement)

After a recovery period of 7 days, the iPSC—CMs are ready for intracellular delivery. As iPSC lines from different donors can show some inter-line variability, we recommend optimizing the photoporation delivery efficiency in your iPSC—CMs using model macromolecules, such as FITC-labelled dextran.

## AuNP-sensitized photoporation

Materials and equipment

- LumiPore™ photoporation platform (Trince, Belgium)
- 0.06 µm AuNP stock with polymer diallyldimethylammonium chloride (PDDAC)-coating (Trince, Belgium)
- FD10 stock (50 mg/ml in PBS) (Sigma-Aldrich cat. no. FD10S)
- FD150 stock (50 mg/ml in PBS) (Sigma-Aldrich cat. no. FD150S)
- PBS (no calcium or magnesium) (Biowest, cat. no. L0615–500)
- Cardio culture medium (see above)
- Microcentrifuge tubes
- Vortex

(1) Preheat the cardio culture medium
(2) Prepare the AuNP dilution in cardio culture from the AuNP stock. We recommend starting with a concentration gradient (1 × 10^7^, 2 × 10^7^, 4 × 10^7^, 8 × 10^7^, 16×10^7^ AuNPs/mL) to find the optimal condition for your cells.
(3) For each concentration, prepare one microcentrifuge tube with the correct amount of medium (for 96 well format: 100 µl per well) + 10 % for pipetting errors
(4) Take the correct amount of AuNP stock and transfer it to the assigned microcentrifuge tube (**mix thoroughly after adding the AuNPs by vortexing**)
(5) Remove the culture medium from the selected wells with cells
(6) Transfer the AuNP dilutions (100 µl/well) to the assigned wells (**mix by resuspending before transferring**)
(7) Incubate the cells for 30 min at 37 °C, 5 % CO_2_, 19 % O_2_ to allow the AuNPs to adhere to the cell membranes
(8) Near the end of the 30 min, turn on the Lumipore device to allow the laser to heat up
(9) Prepare a dilution of FITC-labelled dextran (or your compound of interest) in cardio culture medium (FD10 and FD150 should be diluted to 2 mg/ml)
(10) After the 30 min incubation, wash all cells once with 100 µl PBS to remove unbound AuNPs
(11) Add 50 µl of fresh cardio culture medium with the compound of interest to the wells and fresh cardio culture medium to the non-treated control (NTC) wells. Cells are now ready to be irradiated
(12) Insert the plate into the Lumipore and select the laser fluence of interest and irradiation pattern for each specific well. Screening different laser fluences can be part of the optimization procedure

After photoporation, we recommend checking the delivery efficiency and viability of your cells as explained below.

## Intracellular delivery efficiency quantification by flow cytometry

Materials and equipment

- Flow cytometer
- TO-PRO-3 (Invitrogen, cat.no. T3605)
- Flow buffer (0.1 % Sodium Azide (Sigma-Aldrich, cat. no. 71,289) and 1 % Bovine Serum Albumin (Sigma-Aldrich, cat. no. A9418) diluted in PBS)
- Multi Tissue Dissociation kit 3 (Miltenyi Biotec, cat. no. 130–110–204)
- U-bottom 96 well plate (VWR, cat. no. 734–2328)

(1) Preheat a sufficient volume of the Multi Tissue Dissociation kit 3 enzyme and cardio culture medium
(2) Remove all medium and wash the wells with 100 µl cardio culture medium twice to remove any undelivered cargo molecules (e.g. FITC-dextrans)
(3) Wash wells with 100 µl PBS twice
(4) Add 50 µl of the enzyme mix (Multi Tissue Dissociation kit 3) to all wells
(5) Incubate the well plate for 10 min at 37 °C, 5 % CO_2_, 19 % O_2_. **Check cell detachment and single cell formation under the microscope.** Gently resuspend up to 5 times to obtain a single cell population
(6) Add 100 µl cardio culture medium to all wells to inactivate enzyme activity
(7) Transfer the content of the wells to a U-bottom plate (**pipet up and down first**)
(8) Centrifuge the plate at 500 g for 5 min. *Check pellet after centrifugation*
(9) In the meantime, prepare the TO-PRO-3 solution (1/1000) in flow buffer
(10) Gently remove the supernatant after centrifugation
(11) Resuspend the pellet with 100 µl flow buffer containing the live cell-impermeable TO-PRO-3 dye, in order to exclude the dead cell population in the flow cytometry analysis
(12) Proceed with flow cytometry analysis (FD10 and FD150 – 488 nm laser, 525/50 nm bandpass filter, TO-PRO-3 – 640 nm laser, 667/30 nm bandpass filter)

## Cardiomyocyte viability assay

Materials and equipment

- CellTiter-Glo® Reagent (CTG-reagent) (Promega, cat. No. G7570)
- Glomax® microplate reader (Promega, Belgium)
- Opaque 96 well plate (Greiner Bio-one, cat. No. 655,075)
- Shaker platform
- Aluminum foil

(1) After photoporation, incubate the plate for 2 h at 37 °C, 5 % CO_2_, 19 % O_2_ to let the cells recover
(2) After 2 h, remove the medium of the wells
(3) Add 100 µl of fresh cardio culture medium to the wells

**Shield plate from light for remainder of experiment**

(4) Add 100 µl of the CTG-reagent to all wells, including the blanks
(5) Cover the plate with aluminum foil
(6) Put plate on the shaker for 10 min at 125 rpm
(7) Pipet 100 µl of every well into a new well in the opaque 96 well plate
(8) Cover immediately with tin foil
(9) Let rest for 5–10 min at room temperature
(10) Measure luminescent signal using the Glomax® microplate reader (detection range: 350 to 650 nm)
(11) Calculate the cell viability of each condition as a percentage of luminescence versus the NTC

## Method validation

**Fig. 1:**
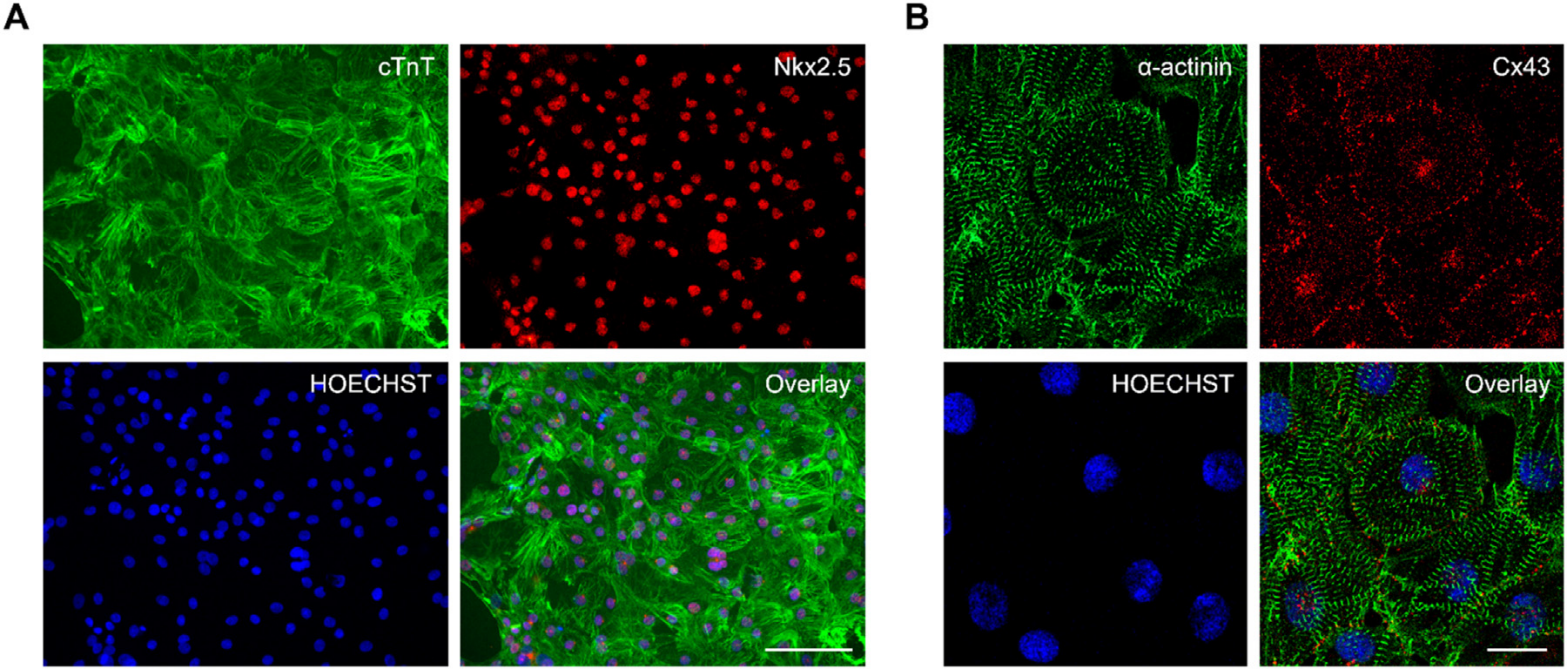
Immunocytochemistry images of the differentiated iPSC-derived cardiomyocytes. Cardiomyocyte identity was confirmed by expression of the specific markers (**A)** TNNT2 in green, Nkx2.5 in red (image acquired using EVOS FL cell imaging microscope (Thermo Fisher)), and (**B)** α-actinin in green and Cx43 in red (image acquired using Zeiss LSM900 confocal microscope). Nuclei were labelled with HOECHST. Scalebar indicates 100 µm in (**A)** and 20 µm in (**B)**.

To validate the described method, iPSCs (described in [12]) were differentiated to cardiomyocytes by addition of small molecules (Supplement 1). After the temporal modulation of the Wnt pathway, the differentiated cardiomyocytes were passaged to coverslips, after which the cardiomyocyte purity and phenotype was analyzed on day 30 after start of differentiation. Immunocytochemistry staining shows cardiomyocytes expressing cardiac-specific troponin T (cTnT), and transcription factor Nkx2.5 (Fig. 1**A**). All nuclei expressed the cardiac transcription factor Nkx2.5, indicating a pure cardiomyocyte population. Furthermore, the sarcomeres stained by α-actinin display clear striations and a well-organized contractile apparatus (Fig. 1**B**). The average distance between subsequent sarcomeres, an indicator of cardiomyocyte maturity, was 1.92 ± 0.09 µm according to image analysis (*n* = 45), which is well in the normal range. Connexin-43 (Cx43), an important gap junction protein, was expressed and localized at the cell-cell borders, indicating intricate electrical connections between adjacent cells (Fig. 1**B**).

**Fig. 2:**
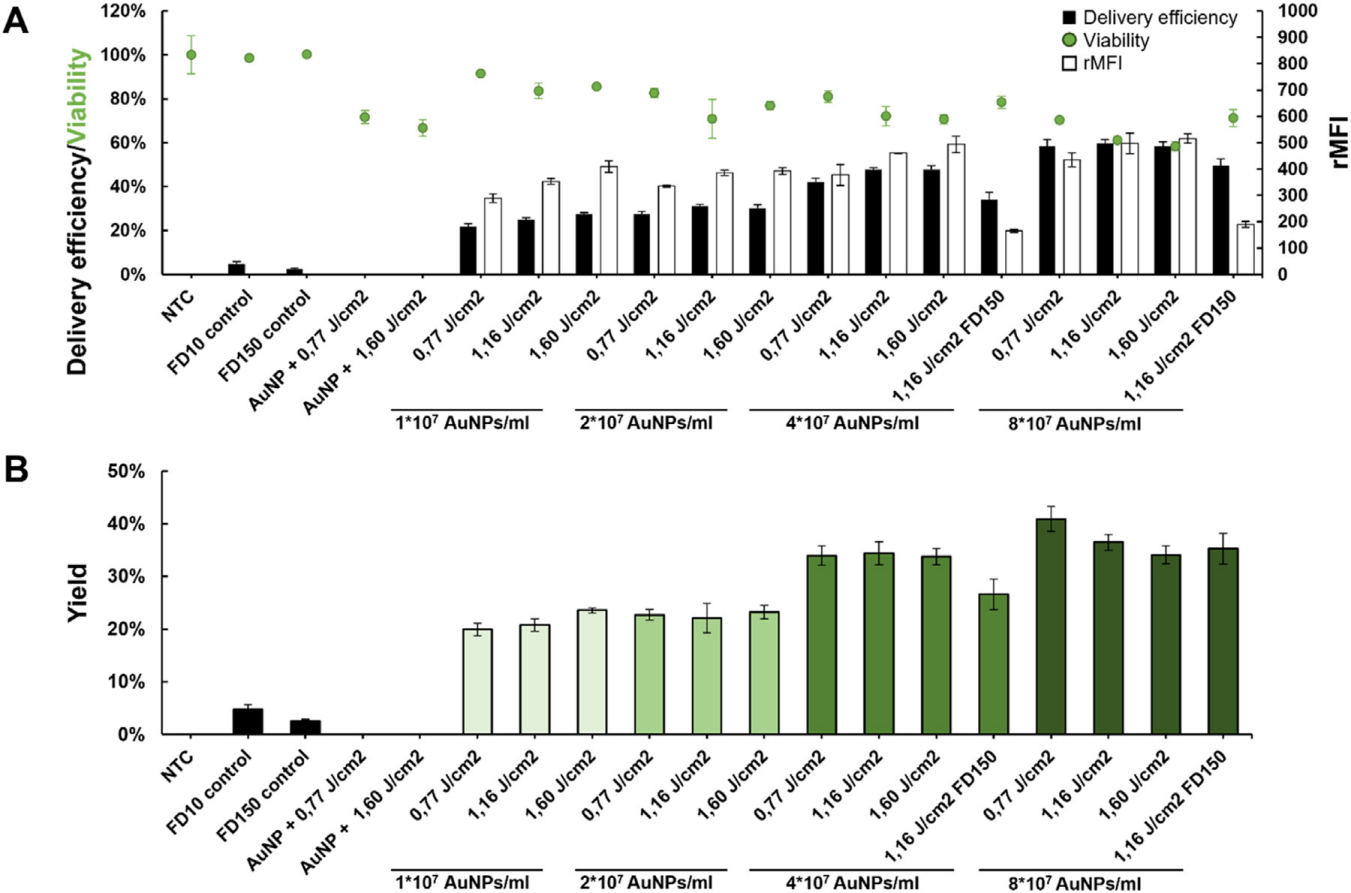
Intracellular delivery of FD10 in iPSC-derived cardiomyocytes with increasing AuNP concentrations (1 × 10^7^, 2 × 10^7^, 4 × 10^7^, 8 × 10^7^ AuNPs/ml) and laser fluences (0,77 J/cm^2^; 1,16 J/cm^2^; 1,60 J/cm^2^). FD150 delivery was performed exclusively using the highest AuNP concentrations (4 × 10^7^ and 8 × 10^7^ AuNPs/ml) and laser fluence 1,16 J/cm². **A)** Delivery efficiency as quantified by flow cytometry is given as a percentage in the black bars. The relative mean fluorescence intensity (rMFI) is indicated in the white bars, with values corresponding to the right axis. Viability was quantified using the CellTiterGlo® assay, and is indicated by the green dots. **B)** Yield of the intracellular delivery, which was calculated by multiplying the delivery efficiency and viability. Error bars indicate the standard deviation, number of repeats (n) = 3.

For convenient optimization of photoporation conditions, iPSC—CMs were seeded in a 96-well plate, and subsequently photoporated using FITC-labelled dextran of two sizes: 10 kDa and 150 kDa. Increasing amounts of AuNPs (1 × 10^7^, 2 × 10^7^, 4 × 10^7^, 8 × 10^7^ AuNPs/ml) were added to the cells, which upon laser irradiation induce more and more transient pores in the iPSC—CM membranes. Three laser fluences (0,77 J/cm^2^; 1,16 J/cm^2^; 1,60 J/cm^2^) were included to find an optimal balance between delivery efficiency and viability. Relevant controls, including the non-treated control (NTC), FD10/FD150 control (addition of FD10/FD150 without photoporation to check for passive cargo uptake by endocytosis) and AuNP + laser control (photoporation without FD10/150 cargo to assess toxicity by photoporation alone) were incorporated. Delivery efficiency was quantified by flow cytometry (MACSQuant 16, Miltenyi Biotec), to determine the percentage of FD10/FD150 positive single cells in the viable cell population (TO-PRO-3 negative) (Fig. 2A). Also, the relative mean fluorescence intensity (rMFI) was determined, by calculating the mean fluorescence intensity (MFI) of the FD10 or FD150 positive cells relative to the MFI of the NTC. This value gives an indication of the amount of FITC-dextran per cell.

**Fig. 3:**
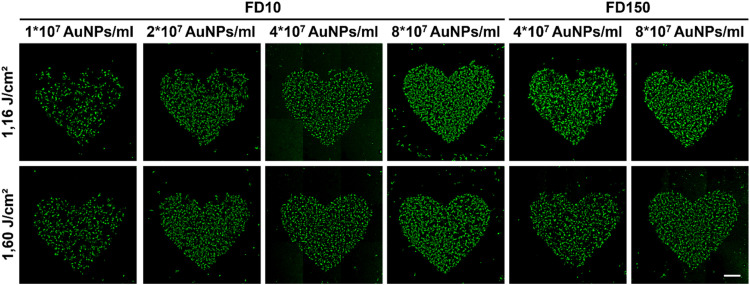
Spatially-selective intracellular delivery of FD10 and FD150 in iPSC-derived cardiomyocytes with increasing AuNP concentrations (1 × 10^7^, 2 × 10^7^, 4 × 10^7^, 8 × 10^7^ AuNPs/ml) and laser fluences (0,77 J/cm^2^; 1,16 J/cm^2^; 1,60 J/cm^2^). A heart-shaped pattern was irradiated instead of the whole well. Images were stitched and acquired using a Zeiss LSM900 confocal microscope. Scalebar indicates 500 µm.

Higher AuNP concentrations generally yielded higher FD10 delivery efficiencies (up to 59,7 % ± 1,8 %) and rMFI values, at the cost of a lower viability. Increasing laser fluences also led to higher delivery efficiencies. The same trend was observed for the FD150 cargo, with a 15 % delivery increase in the 8 × 10^7^ AuNP condition compared to the 4 × 10^7^ AuNP condition, with a minimal loss in cell viability (−8 %). To comprehensively account for all measurements and determine the optimal setting, we calculated the yield for each experimental condition and control group, combining the percentage of FITC-positive cells and viable cells. Using this calculation, we observed that a concentration of 8 × 10^7^ AuNPs/ml combined with a laser fluence of 0,77 J/cm² achieved the highest yield (40,9 %) (Fig. 2B). To demonstrate the possibility for spatial delivery, a heart shape was photoporated in the 96 wells. Excellent spatial resolution was observed in all conditions, expectedly with more cells carrying the FD10/150 cargo in the higher AuNP concentrations and laser fluences (Fig. 3). Some uptake outside the region of interest can be seen in a small percentage of isolated cells, possibly due to permeabilization of apoptotic cells.

## Ethics statements

Stem cell experiments were conducted with the approval of the Ghent University ethics committee: (UZG 2017/0855)

## CRediT authorship contribution statement

**Laurens Léger:** Investigation, Conceptualization, Formal analysis, Methodology, Visualization, Writing – original draft. **Chloë De Clercq:** Investigation, Formal analysis, Methodology, Validation, Writing – original draft. **Jeffrey Aalders:** Investigation, Writing – review & editing. **Kiara Van Acker-Verberckt:** Investigation. **Kevin Braeckmans:** Conceptualization, Funding acquisition, Methodology, Supervision, Writing – review & editing. **Jolanda van Hengel:** Conceptualization, Funding acquisition, Methodology, Supervision, Writing – review & editing.

## Declaration of competing interest

The authors declare the following financial interests/personal relationships which may be considered as potential competing interests:

KB declares financial interest in the company Trince which markets the LumiPore photoporation technology

## Data Availability

Data will be made available on request.

## Appendix Supplementary materials

Image, application 1
