# Supplementary material for: Photoporation-mediated spatial intracellular delivery of stem cell-derived cardiomyocytes
Source: MethodsX. 2024 Jan 4;12:102548. doi: 10.1016/j.mex.2024.102548 (PMC10825475; doi:10.1016/j.mex.2024.102548)
Supplement: Supplementary file 1 [file mmc1.docx]

**Supplementary material *and/or* additional information [OPTIONAL]**

**iPSC culturing and cardiac differentiation**

The human induced pluripotent stem cell (iPSC) line ‘UGENT001-A-1’^12^ (https://hpscreg.eu/cell-line/UGENTi001-A-1) was cultured feeder-free in Essential 8™ medium (Life Technologies, cat no. A1517001) supplemented with Penicillin/Streptomycin (100 u/ml Penicillin and 100 µg/ml Streptomycin, Life Technologies, cat no. 15140-122) on Geltrex™ coating (Life Technologies, cat no. A1413302) in 12-well CELLSTAR® culture plates (Greiner Bio One, cat no. 657160 and cat no. 665180). The iPSC cultures were maintained at 37 °C, 5% CO_2_ and 5% O_2_.

To obtain iPSC-derived cardiomyocytes, iPSCs were passaged as single cells using TrypLE Select (1X) (Life Technologies, cat. no. 12563011) and seeded onto 12-well plates coated with Geltrex^TM^ at a density of 21,500 cells/cm2. To enhance cell survival during the initial 24 hours post-passage, Revitacell^TM^ (Life Technologies, cat no. 12563-011), a rock inhibitor, was added to the E8 medium at a ratio of 1:100. Two days after passaging, the cardio differentiation medium (composed of RPMI1640 with GlutaMAX and HEPES (Life Technologies, cat. no. 72400-021), 0.5 mg/ml human recombinant albumin (Sigma, cat. no. A9731), and 0.2 mg/ml L-ascorbic acid 2-phosphate (Sigma, cat. no. A8960) was supplemented with 4 µM of the GSK-3 inhibitor CHIR99021 (Calbiochem, cat. no. 361559) for a duration of 48 hours. Subsequently, 5 µM of the Wnt Antagonist IWP-2 (Calbiochem, cat. no. 681671), diluted in the cardio differentiation medium, was introduced. Following another 48 hours, the medium was transitioned to cardio differentiation medium without any small molecules. On the 6th day post-differentiation induction, the cardio differentiation medium was renewed for an additional 48 hours before switching to cardio culture medium, which consisted of RPMI 1640 with GlutaMAX and HEPES, supplemented with a 1:50 ratio of B27 supplement with insulin (Life Technologies, cat. no. 17504-044). This medium switch was accompanied by a change in oxygen concentration during incubation (37°C, 5% CO2 and 19% O2). The cardio culture medium was replenished every two days. Visible contractions typically commenced around day 10 to 12. By day 14, the differentiation process was considered successful when approximately 90% of the culture displayed visual beating in a monolayer.
